# Recurrence of crystalline nephropathy after kidney transplantation in APRT deficiency and primary hyperoxaluria

**DOI:** 10.1186/s40697-015-0069-2

**Published:** 2015-09-15

**Authors:** Guillaume Bollée, Pierre Cochat, Michel Daudon

**Affiliations:** Division of Nephrology and Research Centre of the Centre Hospitalier de l’Université de Montréal and Université de Montréal, Montréal, Québec Canada; Centre de Référence des Maladies Rénales Rares, Hospices Civils de Lyon and Université Claude-Bernard Lyon 1, Lyon, France; Service d’Explorations Fonctionnelles, AP-HP, Hôpital Tenon, Paris, France; Division of Nephrology, Hôpital Notre Dame, 1560 Sherbrooke Street East, Montreal, QC H2L 4 M1 Canada

## Abstract

**Purpose of review:**

To provide transplant physicians with a summary of the pathogenesis and diagnosis of adenine phosphoribosyl transferase (APRT) deficiency and primary hyperoxaluria and, focussed on kidney transplantation, and to discuss interventions aimed at preventing and treating the recurrence of crystalline nephropathy in renal transplant recipients.

**Source of information:**

Pubmed literature search.

**Setting:**

Primary hyperoxaluria and APRT deficiency are rare inborn errors of human metabolism. The hallmark of these diseases is the overproduction and urinary excretion of compounds (2,8 dihydroxyadenine in APRT deficiency, oxalate in primary hyperoxaluria) that form urinary crystals. Although recurrent urolithiasis represents the main clinical feature of these diseases, kidney injury can occur as a result of crystal precipitation within the tubules and interstitium, a condition referred to as crystalline nephropathy. Some patients develop end-stage renal disease (ESRD) and may become candidates for kidney transplantation*.* Since kidney transplantation does not correct the underlying metabolic defect, transplant recipients have a high risk of recurrence of crystalline nephropathy, which can lead to graft loss. In some instances, the disease remains undiagnosed until after the occurrence of ESRD or even after kidney transplantation.

**Key messages:**

Patients with APRT deficiency or primary hyperoxaluria may develop ESRD as a result of crystalline nephropathy. In the absence of diagnosis and adequate management, the disease is likely to recur after kidney transplantation, which often leads to rapid loss of renal allograft function. Primary hyperoxaluria, but not APRT deficiency, becomes a systemic disease at low GFR with oxalate deposition leading to malfunction in non-renal organs (systemic oxalosis). We suggest that these diagnoses should be considered in patients with low glomerular filtration rate (GFR) and a history of kidney stones. In APRT deficiency, stones may be confused with uric acid stones, unless specialized techniques are used (infrared spectroscopy or X-ray crystallography for urinary crystals or stone analysis; Fourier transform infrared microscopy for crystals in kidney biopsy). Where these are unavailable, and for confirmation, the diagnosis can be made by measurement of enzyme activity in red blood cell lysates or by genetic testing. In patients with primary hyperoxaluria, levels of urinary and plasma oxalate; and the presence of nearly pure calcium oxalate monohydrate in stones, which often also have an unusually pale colour and unorganized structure, increase diagnostic suspicion. Molecular genetic testing is the criterion measure. Lifelong allopurinol therapy, with high fluid intake if appropriate, may stabilize kidney function in APRT deficiency; if ESRD has occurred or is near, results with kidney transplantation after initiation of allopurinol are excellent. In primary hyperoxaluria recognized before ESRD, pyridoxine treatment and high fluid intake may lead to a substantial decrease in urinary calcium oxalate supersaturation and prevent renal failure. In non-responsive patients or those recognized later in their disease, liver transplantation cures the underlying defect and should be considered when the GFR falls below 30 ml/min/1.73 m^2^; in those which or near ESRD, liver transplantation and intensive dialysis before kidney transplantation may be considered to reduce the total body oxalate burden before kidney transplantation.

**Limitations:**

The availability of diagnostic tests varies between countries and centres. Data on long term outcomes after kidney transplantation are limited, especially for APRT deficiency patients.

**Implications:**

Increasing transplant physicians knowledge of APRT deficiency and primary hyperoxaluria should enable them to implement adequate diagnostic and therapeutic interventions, thereby achieving good outcomes after kidney transplantation.

## RÉSUMÉ

**Objet de l’étude:**

Fournir aux médecins transplantologues un résumé de la pathogénie, de même que les diagnostics de déficit en adénine phosphoribosyl transférase (APRT) et d’hyperoxalurie primaire, dans le contexte de la greffe rénale, ainsi que discuter des interventions visant à prévenir et à traiter la récurrence d’une néphropathie cristalline chez les greffés du rein.

**Source d’information:**

Recherche de documentation dans Pubmed.

**Contexte:**

L’hyperoxalurie primaire et déficit en APRT sont des maladies enzymatiques humaines. Ces maladies se caractérisent par la surproduction et l’excrétion urinaire d’éléments (2,8-dihydroxyadénine pour le déficit en APRT et oxalate pour l’hyperoxalurie primaire) qui forment des cristaux urinaires. Bien que l’urolithiase récurrente représente la caractéristique clinique principale de ces maladies, la précipitation des cristaux au niveau tubulo-interstitiel peut entraîner des lésions rénales, un état pathologique appelé néphropathie cristalline. Certains patients développent une insuffisance rénale terminale (IRT) et peuvent devenir candidats à la greffe rénale. Étant donné que la greffe du rein ne corrige pas le défaut métabolique sous-jacent, les greffés présentent un risque élevé de récurrence de néphropathie cristalline, qui peut mener à la perte du greffon. Dans certains cas, la maladie n’est diagnostiquée qu’après l’occurrence de l’IRT, voire à la suite de la greffe du rein.

**Messages clés:**

Les patients souffrant d'un déficit en APRT ou d’hyperoxalurie primaire risquent de développer une IRT engendrée par la néphropathie cristalline. En l’absence d’un diagnostic et d’une gestion adéquate, la maladie réapparaîtra probablement suite de la greffe du rein, ce qui entraînera souvent la perte précipitée de l’allogreffe. L’hyperoxalurie primaire, et non le déficit en APRT, devient une maladie systémique à faible débit de filtration glomérulaire (DFG) caractérisée par des dépôts d’oxalate, et menant à la défaillance d’organes autres que le rein (oxalose systémique). Nous suggérons que ces diagnostics soient priss en considération chez les patients comportant un faible DFG, de même que des antécédents de calculs rénaux. Dans le cas d'un déficit en APRT, les calculs rénaux pourraient être confondus avec des calculs d’acide urique, à moins que ne soient employées des techniques spécialisées (spectroscopie infrarouge ou radiocristallographie pour l’analyse des cristaux urinaires ou des calculs par rayon X; microscopie infrarouge à transformer de Fourier pour les cristaux au cours de la biopsie des calculs). Lorsque possible, et à des fins de confirmation, le diagnostic peut être effectué par la mesure de l’activité enzymatique de la lyse des cellules sanguines rouges ou par le dépistage génétique. Chez les patients atteints d’hyperoxalurie primaire, les taux d’oxalates urinaire et plasmique, de même que la présence d’oxalate calcique monohydrate à l’état presque pur dans les calculs, qui présentent aussi généralement une pâleur et une structure désorganisée, éveillent les soupçons quant au diagnostic. Le dépistage génétique moléculaire constitue le critère de mesure. Un traitement d’allopurinol à vie, ainsi qu’un apport élevé en liquides si nécessaire, peut stabiliser la fonction rénale en situation de déficit en APRT. Si l’IRT s’est produite ou approche, les résultats de la greffe du rein à la suite du début du traitement à l’allopurinol sont excellents. Dans le cas d’une hyperoxalurie détectée avant l’IRT, un traitement à la pyridoxine et un apport élevé en liquides peuvent mener à une baisse substantielle de la sursaturation en oxalate calcique urinaire et ainsi, prévenir l’insuffisance rénale. Chez les patients qui ne répondent pas au traitement ou ceux chez qui la maladie est détectée tardivement, la transplantation hépatique enraye le défaut sous-jacent, et devrait être envisagée si le DFG chute en dessous de 30 ml/min/1,73 m^2^. Chez les patients en IRT ou près de l’être, la transplantation hépatique et la dialyse intensive la précédant sont à envisager afin de réduire la charge corporelle totale d’oxalate avant la transplantation hépatique.

**Limites de l’étude:**

L’accessibilité à des tests de dépistage varie selon les pays et les unités. Les données sur les résultats à long terme de la greffe rénale sont limitées, particulièrement en ce qui concerne les patients présentant un déficit en APRT.

**Conséquences:**

Enrichir les connaissances des transplantologues au sujet du déficit en APRT et de l’hyperoxalurie primaire devrait leur permettre de mettre en œuvre des interventions diagnostiques et thérapeutiques adéquates, et ainsi d’obtenir de bons résultats à la suite des greffes rénales.

## Why is this review important?

Transplant physicians need to be aware of the risk of relapse of crystalline nephropathy in kidney transplant recipients with APRT deficiency or primary hyperoxaluria.

## What are the key messages?

Clinicians should consider APRT deficiency and primary hyperoxaluria in patients with low GFR and stones. Though there are findings in urine, biopsy and stone analysis that may be suggestive, not all patients will have urine, biopsies or stones available for assessment, and special tests, not widely available, are needed in each of these evaluations to effectively rule in or rule out the disorders. Diagnostic testing by red cell APRT assay for APRT deficiency and genetic testing in either condition can be performed at any stage of chronic kidney disease (CKD).

Early recognition of the disease and specific therapeutic interventions are of prime importance to prevent relapse of crystalline nephropathy, which can lead to rapid kidney allograft failure.

## Introduction

Adenine phosphoribosyl transferase (APRT) deficiency and primary hyperoxaluria are rare inborn errors of human metabolism. These diseases have in common the overproduction of a metabolic compound, 2,8 dihydroxyadenine (DHA) in APRT deficiency, and oxalate in primary hyperoxaluria, which is excreted by the kidney and precipitates in the urine, leading to the formation of urinary crystals and kidney stones. Although recurrent urolithiasis represents the main clinical feature of these diseases, kidney damage can occur as a result of tubular toxicity and precipitation of crystals within the tubules and interstitium, a condition referred to as crystalline nephropathy. Recent studies provide novel insights into the mechanisms of crystal-related kidney inflammation and injury [1]. In severe cases of primary hyperoxaluria or APRT deficiency, disease progression can lead to end-stage renal disease (ESRD) and patients become candidates for kidney transplantation*.* Since kidney transplantation does not correct the underlying metabolic defect, transplant recipients have a high risk of recurrence of crystalline nephropathy, which can cause loss of renal allograft function. Preventing such an outcome requires diagnosis and specific interventions. Thus, transplant nephrologists and urologists should have sufficient knowledge of APRT deficiency and primary hyperoxaluria to enable them to suspect, diagnose and manage these diseases in the context of kidney transplantation. The purpose of this review is to provide concise information about the pathogenesis, clinical presentation and diagnosis of APRT deficiency and primary hyperoxaluria and to discuss interventions aimed at preventing and treating the recurrence of crystalline nephropathy. Readers looking for more in-depth information on genetics, mechanisms and phenotype of APRT deficiency or primary hyperoxaluria are referred to recent reviews on these topics [2–5].

## From metabolic deficiency to kidney injury and transplantation

### Mechanisms of disease and clinical presentation

#### APRT deficiency

APRT deficiency (number 614723 in the Online Mendelian Inheritance of Man [OMIM] database), is a rare inherited disease caused by a complete deficiency of the APRT enzyme, produced by mutations in the *APRT* gene (Table 1). The disease is of autosomal recessive inheritance and individuals carrying only one mutant *APRT* allele (heterozygotes) are asymptomatic despite lower-than-normal levels of enzyme activity. Two types of APRT deficiency have been described, based on the level of APRT enzyme activity measured *in vitro* in cell lysates. Type 1 is characterized by complete deficiency *in vitro*, whereas APRT activity is low, but still detectable, in type 2. Type 2 has been reported almost exclusively in Japanese, whereas type 1 has been observed in other ethnic groups [6–8]. Noteworthy, in both types APRT activity is nil *in vivo* and the clinical manifestations are similar [2].

**Table 1:** Hallmarks of APRT deficiency and primary hyperoxaluria type 1. (APRT: Adenine phosphoribosil transferase, AGT: alanine-glyoxylate aminotransferase

	APRT deficiency	Primary hyperoxaluria type 1
Inheritance	autosomal recessive	autosomal recessive
Enzyme deficiency/tissue expression	APRT/ubiquitous	AGT/liver
Disease causing end-product	2,8 dihydroxyadenine	oxalate
Urinary crystals	2,8 dihydroxyadenine	calcium oxalate monohydrate
Age at diagnosis	from infancy to adulthood	from infancy to adulthood
Clinical manifestations		
- kidney stones	yes, radiolucent	yes, radiopaque
- nephrocalcinosis	no	yes
- crystalline nephropathy	yes	yes
- extrarenal manifestations	no	systemic, especially bones, blood vessels, heart, retina
Reccurrence on renal allograft	yes	yes

Since APRT represents the only pathway for adenine metabolism, the complete loss of APRT activity results in the accumulation of adenine, which is then converted to 8-hydroxyadenine and then to 2,8-dihydroxyadenine (DHA) by the xanthine dehydrogenase enzyme. DHA is cleared by the kidney and excreted in large quantities in urine [9]. Since DHA is insoluble in urine, it precipitates in the form of crystals that can aggregate and eventually lead to the formation of kidney stones. DHA crystals can also precipitate within the renal tubules and interstitium and cause severe injury to the native as well as the transplant kidney [6, 8, 10]. We recently proposed the term “DHA nephropathy” for the kidney disease caused by DHA crystals [2]. The disease is not caused by the absence of functional APRT enzyme in the kidney, but rather to systemic deficiency. One important implication of this is that the metabolic disorder is not corrected by kidney transplantation. Although the APRT enzyme is ubiquitously expressed and organs other than the kidney are exposed to elevated systemic levels of DHA, APRT deficiency is not known to cause extrarenal symptoms.

Although the worldwide prevalence of APRT deficiency is largely unknown, it has been estimated to be 1/100,000 in Caucasian population [11, 12]. The prevalence is higher in some countries, especially Japan, Iceland and France, due to the frequency of certain mutations in these areas [6–8]. In many countries, only few cases have been reported so far, which may reflect underdiagnosis [13].

Recurrent kidney stones are the most common presentation of APRT deficiency [7, 14]. The age at onset of symptoms varies from infancy to later than the fourth decade [2, 6, 8, 15]. Noteworthy, DHA stones are radiolucent and often mistaken for uric acid stones. Crystalline nephropathy secondary to DHA deposition into the kidney mainly occurs in adults with a history of repeated episodes of kidney stones, in whom the disease has remained undiagnosed and untreated for years [2]. However, crystalline nephropathy and progressive worsening of renal function patients may occur in patients who had only a few, or even no episodes of kidney stones [16, 17]. In the absence of appropriate therapy, kidney injury may progress over time and lead to ESRD. In Japanese and European cohorts, about 10 % of patients had reached ESRD prior to diagnosis of APRT deficiency, with diagnosis being made after kidney transplantation in most cases [6–8]. This is particularly regrettable, given the efficiency of allopurinol to treat APRT deficiency, as discussed below.

#### Primary hyperoxaluria

Primary hyperoxalurias are a group of autosomal recessive diseases characterized by the overproduction of oxalate resulting from enzymatic defects in glyoxylate metabolism. Three forms of primary hyperoxaluria have been identified, with type 1 (OMIM 259900) being by far the most common and also the most severe. Type 1 accounts for 80 % of cases of primary hyperoxaluria and has a prevalence of 1 to 3.4/1,000,000 in North America and Europe [18, 19]. Primary hyperoxaluria type 1 (Table 1) is caused by mutations in the *AGXT* gene encoding the alanine-glyoxylate aminotransferase (AGT), a pyridoxine-dependent liver enzyme that catalyzes the conversion of glyoxylate to glycine. AGT deficiency results in the accumulation of glyoxylate, and overproduction of oxalate and glycolate. Primary hyperoxalurias type 2 (OMIM 260000) and type 3 (OMIM 613616) are respectively caused by a deficiency of glyoxylate reductase-hydroxypyruvate reductase (GRHPR) and 4-hydroxy-2-oxo-glutarate aldolase (HOGA) and each account for approximately 10 % of all primary hyperoxalurias [5, 18]. These enzymes are involved in the reduction of glyoxylate to glycolate (GRHPR) and metabolism of hydroxyproline (HOGA). The excess oxalate produced by the liver is then excreted in the urine, where it precipitates in the form of calcium oxalate monohydrate (whewellite) crystals. This results in the formation of kidney stones and to crystal deposition in the renal parenchyma, leading to nephrocalcinosis, chronic kidney disease (CKD) and eventually ESRD. The clinical presentation is highly variable, ranging from recurrent nephrolithiasis and renal failure during childhood to occasional kidney stones in adulthood. Calcium oxalate stones are radiopaque. Similarly to APRT deficiency, the disease is under recognized and 20 to 35 % of patients have ESRD at the time of diagnosis [19, 20]. Renal failure commonly occurs in patients with primary hyperoxaluria type 1 or 2, but is exceptional in patients with type 3, which has the least severe course [21]. Overall, many patients with primary hyperoxaluria eventually require renal replacement therapy and kidney transplantation is considered in most of them. When the glomerular filtration rate (GFR) decreases below 40 ml per minute per 1.73 m^2^, the kidney becomes unable to eliminate the oxalate load. As a result of this, plasma oxalate levels rise and systemic deposition of calcium oxalate ensues [19]. This complication, referred to as systemic oxalosis, can involve almost any organ, especially the bones, blood vessels, heart, and retina, thereby causing debilitating and life threatening complications [5, 22]. As discussed below, liver transplantation is the only therapeutic option with the potential to correct the enzymatic deficiency. The only exception to this is that some mutations in primary hyperoxaluria type 1 (Gly170Arg and Phe152Ile) may be associated with a good response to pyridoxine therapy [23, 24].

### The issue of disease recurrence after kidney transplantation

As discussed above, ESRD can occur in the course of APRT deficiency or primary hyperoxaluria, in young adults as well as in pediatric patients. Obviously kidney transplantation is often considered in these patients. Although successful transplantation may restore renal function, kidney transplantation alone will not correct the enzymatic deficiency that caused the problem in the first place. If no specific therapy aimed at correcting the overproduction of DHA or oxalate is undertaken, kidney stones and crystal deposition are likely to recur and jeopardize the renal allograft.

#### Post transplantation recurrence in APRT deficiency

Nearly twenty cases of DHA nephropathy relapse after kidney transplantation have been reported since the first description by Gagné *et al* in 1994 [6, 25–31]. Relapses appear almost exclusively in patients in whom the diagnosis of APRT deficiency has not been made before transplantation and who are therefore not receiving allopurinol. In the majority of cases reported, the disease was only recognized during biopsy evaluation of kidney allograft dysfunction, which was found to be secondary to DHA deposits. The presentation and severity of the recurrence range from primary non-function to progressive worsening of renal function, leading to allograft failure over a period of several months following transplantation [28, 29, 31]. In some patients, the course of the relapse may be mild with deterioration of graft function over several years. Although APRT deficiency mainly recurs in the form of crystalline nephropathy, it may also manifest as kidney stones developing after transplantation [32].

#### Recurrence of primary hyperoxaluria

Similarly to APRT deficiency, diagnosis of primary hyperoxaluria is made in the context of post-transplantation recurrence in roughly 10 % of patients. Kidney transplantation has been performed in patients known to have primary hyperoxaluria, although combined liver-kidney transplantation is the preferred therapeutic option, as discussed later.

After kidney transplantation alone, the allograft is exposed not only to the oxalate produced in excess by the liver, but also to tissue stores of oxalate that are mobilized with return of renal function [33]. Several studies highlighted the poor outcome of isolated kidney transplantation in primary hyperoxaluria type 1. In the 1990 report of the European Dialysis and Transplant Association Registry, which included 98 patients, the 3-year renal survival was 23 % for kidneys from living donor kidneys and 17 % for cadaveric kidneys [34]. Data from the International Primary Hyperoxaluria Registry published in 2010 indicated a 5-year kidney graft survival of 45 % for patients who received a kidney transplant alone (n = 44) [35]. More recently, data from the European Registry showed a graft survival of 46 %, 28 %, and 14 % at 1, 3, and 5 years respectively in 13 children with primary hyperoxaluria who underwent kidney transplantation alone [36]. Early failure of kidney transplantation related to recurrence of oxalate calcium deposition in the renal allograft can also occur in primary hyperoxaluria type 2 [37].

## Diagnosis of aprt deficiency and primary hyperoxaluria in the context of transplantation

### A diagnostic challenge for kidney transplant physicians

Considering the poor outcomes when crystalline nephropathy recurrence after transplantation, it is obviously optimal to recognize APRT deficiency or primary hyperoxaluria as early as possible, in order to implement specific therapies and strategies aimed at lowering DHA or oxalate levels. Not surprisingly, delay in the diagnosis of primary hyperoxaluria diagnosis until after transplantation is associated with a poor outcome [35]. In patients with CKD, APRT deficiency and primary hyperoxaluria are frequently misdiagnosed as non-genetic kidney stones, chronic interstitial nephritis, obstructive nephropathy or hypertensive nephrosclerosis. As a result of this, diagnosis is often delayed, especially in adults. In one study, ESRD was present at the time of diagnosis of primary hyperoxaluria in 26 % of children and in 52 % of adults [19]. In APRT deficiency, several years or even decades may elapse between the onset of symptoms and diagnosis, with ESRD occurring in a substantial proportion of undiagnosed patients [6, 7]. This largely reflects the lack of adequate metabolic screening in patients with recurrent urolithiasis or CKD.

APRT deficiency and primary hyperoxaluria can pose a diagnostic challenge to transplant nephrologists in two situations. First, one patient has ESRD and is evaluated or listed for kidney transplantation. Second, the patient has already received a kidney transplant.

We believe that the possibility of APRT deficiency or primary hyperoxaluria should be questioned in all ESRD patients with a history of urolithiasis unless the nature of the nephropathy and kidney stones has been unambiguously established elsewhere. One pitfall is that some patients may have experienced only a few stone episodes, many years before they developed ESRD. Rarely, there may be no history of urolithiasis prior to ESRD [16, 17]. In addition, the family history is often negative, since APRT deficiency and primary hyperoxaluria are of autosomal recessive inheritance.

**Fig. 1:**
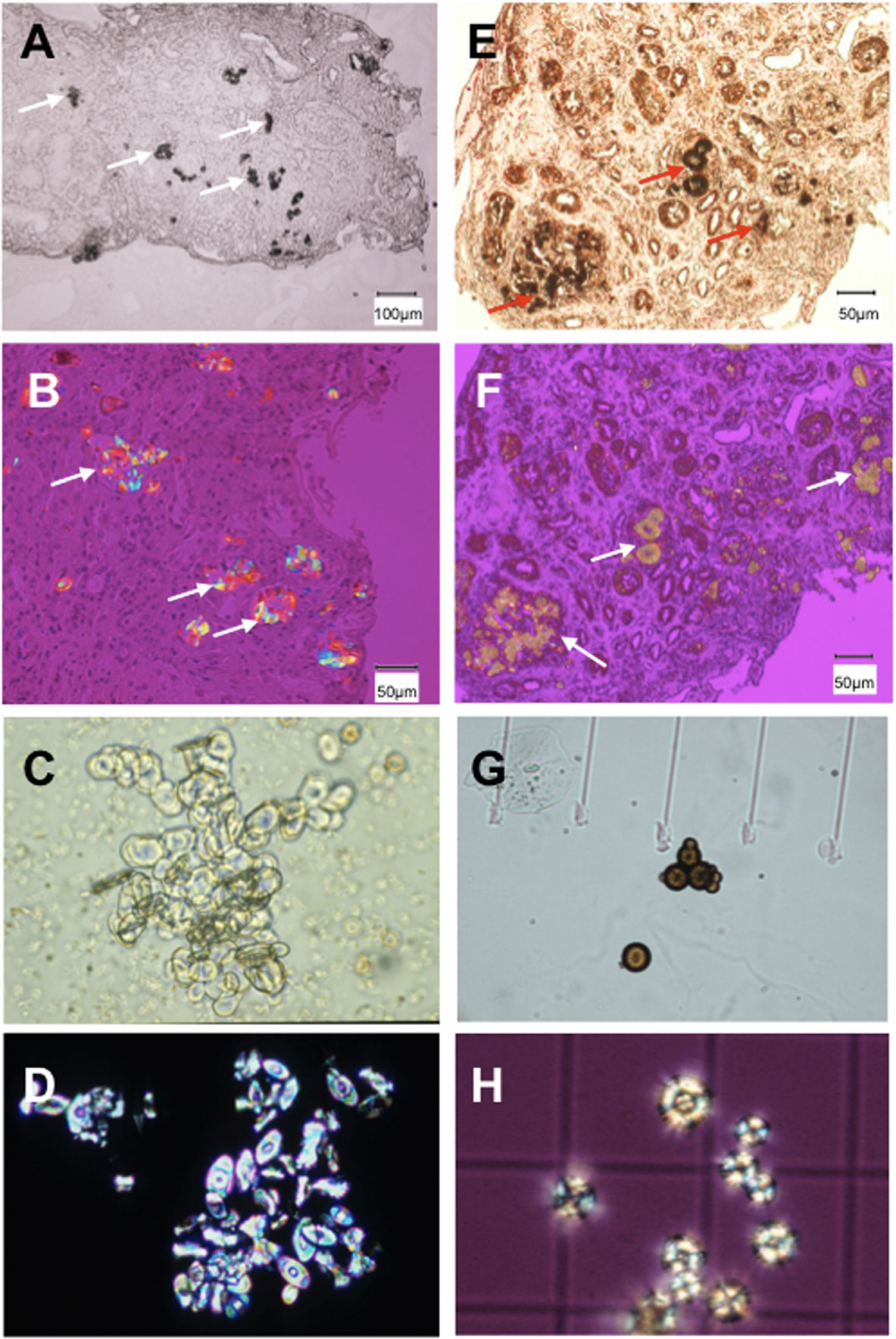
Calcium oxalate (whewellite) and 2,8 dihydroxyadenine (DHA) crystals in kidney biopsy and in the urine. **a** Light microscopy and **b** polarized microscopy of kidney biopsy specimen from a patient with crystalline nephropathy related to primary hyperoxaluria type 1 (arrows: crystal aggregates (scale bar A : 100 μm, B : 50 μm, hematoxylin-eosin-safran staining). Light microscopy aspect of calcium oxalate monohydrate (whewellite) crystals in the urine of a patient with primary hyperoxaluria, under **c** non-polarized and **d** polarized conditions. (400x magnification). **e** Non-polarized and **f** polarized light microscopy of kidney biopsy specimen from a patient with DHA nephropathy in the context of APRT deficiency (arrows: crystal aggregates; scale bar 50 μm, hematoxylin-eosin-safran staining). Urine DHA crystals under **g** non polarized conditions (200x magnification) or **h** polarized light in a patient with APRT deficiency (400x magnification)

A history of urolithiasis or crystals reported in a native or transplant kidney biopsy should lead transplant physicians to consider the possibility of primary hyperoxaluria or APRT deficiency. In some instances, the diagnosis of crystalline nephropathy has not been made despite a kidney biopsy report showing the presence of crystals (Fig. 1). Crystal deposits seen in kidney biopsies are too often misinterpreted as non-specific, which can lead to erroneous diagnosis, such as interstitial nephritis, allograft rejection, or acute tubular necrosis [29]. Of note, crystals may be few despite significant tubulointerstitial changes [28]. We strongly recommend that patients with crystals seen in a transplant or native kidney biopsy always be investigated for these diagnoses.

Several tests can be used to confirm or eliminate the diagnosis of APRT deficiency or primary hyperoxaluria in the context of dialysis or kidney transplantation. Diagnostic tests for APRT deficiency and primary hyperoxaluria type 1 are summarized in Table 2.

**Table 2:** Summary of diagnostic tests for APRT deficiency and primary hyperoxaluria in the context of dialysis or kidney transplantation. (APRT: Adenine phosphoribosil transferase, AGT: alanine-glyoxylate aminotransferase, DHA: 2,8 dihydroxyadenine, FTIR: Fourier transform infrared microscopy, CKD: chronic kidney disease, ESRD: end stage renal disease). In bold *: test sufficient for diagnosis if positive

	APRT deficiency	Primary hyperoxaluria type 1	Advantages	Limitations
Kidney stone analysis	DHA*	nearly 100 % whewellite	any stone previously passed can be studied	stone required
			DHA pathognomonic of APRT deficiency	
			morphology and composition (100 % whewellite) suggestive of primary hyperoxaluria	
Crystalluria	DHA*	calcium oxalate (whewellite)	DHA pathognomonic of APRT deficiency	not feasible in anuric patients
			whewellite crystalluria may suggest primary hyperoxaluria (unspecific)	rare false negative in ESRD patients
FTIR/kidney biopsy	DHA*	calcium oxalate (whewellite)	characterization of crystals in kidney biopsy	kdney biopsy required
			highly sensitive and specific	limited availability
Urinary oxalate		>0.7 mmol/1.73 m^2^/day suggests primary hyperoxaluria	availability, easy to perform	no clear cut-off
				may be normal in patients with advanced CKD
Plasma oxalate		>30 to 50 μmol/l suggests primary hyperoxaluria	availability, easy to perform	no clear cut-off
				useless in patients without advanced CKD
Enzyme activity	decreased APRT activity in erythrocyte lysates*	decreased AGT activity in hepatocytes*	highly sensitive and specific	AGT activity: liver biopsy required, superseded by genetic testing
				APRT activity: not reliable after blood transfusion, limited availability
Molecular study	**mutations in** ***APRT*** gene (two alleles)*	**mutations in** ***AGXT*** gene (two alleles)*	identification of 90 % of mutations in APRT gene, >95 % in AGXT gene	cost, whole gene sequencing may be required

### Tests for diagnosis of both APRT deficiency and primary hyperoxaluria

#### Kidney stone analysis

Stone analysis should be done whenever possible and careful attention should be paid to the results of stone analysis that may have been previously done. Whereas the presence of DHA is pathognomonic of APRT deficiency, the absence of this compound in kidney stones theoretically rules out the disease. However, it is important to verify what technique was used for stone analysis, because standard biochemical tests fail to differentiate between uric acid and DHA. It is now recommended that infrared spectroscopy or X-ray crystallography be used for any stone analysis [38]. In primary hyperoxaluria, stones are almost always composed of nearly pure (>95 %) calcium oxalate monohydrate (whewellite). In comparison with other calcium oxalate calculi with similarly high whewellite content, primary hyperoxaluria type 1 stones have a peculiar morphology, with an unusually pale color and an unorganized structure [39].

#### Study of crystals in kidney biopsy or in the urine

Defining the nature of crystals seen on a renal biopsy may represent an important step for diagnosis of crystalline nephropathy. This is particularly true for APRT deficiency, where the presence of DHA crystals is pathognomonic of the disease. It should be emphasized that light and polarized microscopy study alone cannot provide a reliable characterization of crystals in kidney biopsies. Differential diagnosis of crystals in renal biopsy also includes calcium oxalate (associated with primary hyperoxaluria or other conditions), calcium phosphate, uric acid, drugs, and other rare components such as methyl-1 uric acid, opaline silica or calcium carbonate [40]. Due to their rarity, DHA crystals especially are often confused with oxalate or uric acid crystals and the characteristic Maltese-cross pattern is often absent. In one study, the Maltese-cross pattern was observed in only 2 out of 9 allograft biopsies of patients with recurrent DHA crystalline nephropathy [29]. Fourier transform infrared microscopy (FTIR) used in combination with polarizing microscopy is a reliable technique that distinguishes these crystals in kidney biopsy with excellent sensitivity and specificity [40]. Unfortunately, the availability of this technique is limited to a few specialized centres.

It is easier to characterize crystals in the urine than in kidney biopsy. Crystalluria study by light and polarizing microscopy represents a noninvasive, rapid, cheap and efficient diagnostic test. DHA crystals are typically rounded, reddish-brown, with a central Maltese cross pattern under polarized light microscopy (Fig. 1). Infrared spectrophotometry is required to confirm the composition of crystals seen in urine. In general, crystalluria is present in nearly all patients with primary hyperoxaluria or APRT deficiency [2]. However, the diagnostic value of crystalluria may be limited in candidates for kidney transplantation. Obviously, crystalluria cannot be studied in anuric patients. In addition, there is a concern that crystalluria might be negative in some patients with severe kidney impairment, as reported in APRT deficiency [7]. In dialysis patients, therefore, the absence of crystalluria does not rule out the diagnosis. We advise measurement of APRT activity (see below) in patients with crystalline nephropathy when FTIR is not available or crystalluria cannot be studied. In contrast, urine microscopy is useful to detect crystals in kidney transplant recipients if they have allograft function. In APRT deficiency, crystals may be observed in almost all patients not receiving allopurinol [6, 29]. In our experience, the pathognomonic round and reddish-brown DHA crystals may be visualized almost immediately after the initial diuresis has resolved (Fig. 1). In some instances, DHA crystals seen in urine may be dismissed or misclassified as other crystalline species [17, 28].

Contrary to DHA in APRT deficiency, calcium oxalate monohydrate crystals are not pathognomonic and can be observed in conditions other than primary hyperoxaluria. However, due to its strong relation with hyperoxaluria, the presence of calcium oxalate monohydrate crystals (by contrast with calcium oxalate dihydrate), increase the pretest probability of primary hyperoxaluria, especially when abundant in urine. The recognition of calcium oxalate crystals of either type in kidney biopsy or in the urine may be an important step for diagnosis of primary hyperoxaluria (Fig. 1). The presence of calcium oxalate crystals in a native kidney or graft biopsy or heavy crystalluria, which is nearly constant following transplantation [41], should raise suspicion of primary hyperoxaluria and lead to additional diagnostic work-up.

### Specific tests for APRT deficiency

#### Measurement of APRT enzyme activity

Measurement of enzyme activity in red blood cell lysates is a non-invasive and reliable test for diagnosis of APRT deficiency. It is particularly helpful when no kidney stone is available for analysis and crystalluria cannot be studied (eg, patients on dialysis). Measurement of enzyme activity demonstrates abnormally decreased APRT activity in virtually all patients affected (0 % in type 1 and less than 30 % in type 2) [2]. **Since APRT activity is measured in erythrocytes lysates, false normal values may occur following a blood transfusion.** The main downside of APRT activity assay is its availability, which is limited in most countries.

#### Molecular genetic testing

Sequencing of exons and flanking intronic sequences of the *APRT* gene allows identifying about 90 % of mutations [42]. Although the study of crystals or kidney stones and APRT activity assay are usually preferred as primary diagnostic tests, mutation screening may be used as first-line test when other techniques are not feasible or available. APRT deficiency is confirmed when genetic testing demonstrates functionally significant mutations in both alleles. If only one or no mutation is found, other diagnostic methods, especially APRT activity assay, must be considered.

### Specific tests for primary hyperoxaluria

#### Urinary and plasma oxalate

Measurement of oxalate is useful as first-line evaluation for primary hyperoxaluria, although it does not provide a definitive diagnosis, since plasma and urine concentrations show considerable variation [43]. Primary hyperoxaluria is usually associated with an elevated urine oxalate excretion > 0.5 mmol/1.73 m^2^ per day. Although there is no clear cut-off to distinguish between primary and secondary hyperoxaluria, a urine oxalate excretion > 0.7 mmol/1.73 m^2^ per day is suggestive of primary hyperoxaluria [5]. However, measurement of urinary oxalate is of limited value in patients with advanced CKD or in transplant patients with low GFR, since oxalate excretion decreases as the GFR falls. Urinary oxalate levels may be normal or only moderately increased in patients with advanced CKD [44].

In patients with preserved renal function (GFR higher than 45 ml/min per 1.73 m^2^), plasma oxalate measurement is of little interest since plasma levels are still in the normal range. Although there is considerable variation in plasma oxalate levels among patients with decreased kidney function, values higher than 30 to 50 μmol/l are suggestive of primary hyperoxaluria [45]. Although there is no clear cut-off in patients with ESRD, plasma oxalate levels are usually higher in those with primary hyperoxaluria (>60 μmol/l) than in other patients (<60 μmol/l) [46].

#### Molecular genetic testing

Genetic testing has excellent sensitivity and specificity and is now used as first-line test for primary hyperoxaluria. Since type 1 accounts for about 80 % of cases of primary hyperoxaluria, the *AGXT* gene should be screened first [4, 43]. Whole gene sequencing was reported to be successful in identifying both mutations in 98 % of cases [47]. Selected exonic sequencing or targeted mutation testing may be used for first-line screening, although additional sequencing may be necessary [48, 49].

If no mutation is found in the *AGXT* gene, the next step is to search for mutations the *GRHPR* gene (type 2).

#### Measurement of enzyme activity

Liver biopsy for measurement of AGT and GRHPR enzyme activity is no longer routinely used, because of the availability and reliability of genetic testing and the risk of bleeding. Liver biopsy may be useful in unusual circumstances, where no mutation is found despite a phenotype highly suggestive of primary hyperoxaluria type 1 or type 2.

## Prevention and management of recurrence of crystalline nephropathy

### Prevention and management of recurrence of crystalline nephropathy in APRT deficiency

#### Treatment

Life-long treatment with allopurinol is the mainstay of therapy for APRT deficiency. Allopurinol acts by inhibiting the activity of xanthine dehydrogenase, thereby blocking the conversion of adenine into DHA. In most patients, a daily dose of 200-600 mg per day in adults and 5-10 mg/kg per day in children dramatically reduces crystal formation [6, 14]. Febuxostat, another xanthine dehydrogenase inhibiting drug, may be used as alternative to allopurinol in intolerant patients [50].

Along with xanthine dehydrogenase inhibiting therapy, a fluid intake of at least 2.5-3 litres of water per day (for adult patients) should be encouraged unless contraindicated (eg, patients on dialysis). A low purine diet is usually recommended, though how much this decreases DHA excretion is not known. Since DHA, unlike uric acid, remains very insoluble at high pH values, we do not recommend urine alkalinization.

Only a minority of patients treated in this way experience recurrence of kidney stones. In patients with established DHA nephropathy, treatment usually stabilizes kidney function or ameliorates the rate of loss [6, 7]. In several kidney transplant recipients in whom allopurinol therapy was started in the context of recurrence of DHA nephropathy, treatment led to an improvement or stabilization of allograft function [30, 31]. In a recent series, allograft function improved in 7 out of nine patients under allopurinol therapy [29]. The sooner the treatment is initiated, the better are the chances of achieving a good outcome. Allograft dysfunction may be irreversible if severe tubulointerstitial injury was already present when the treatment was started. Although evidence is very limited, we suggest that dialysis patients awaiting kidney transplantation be treated with allopurinol, in order to achieve metabolic control before the new kidney is implanted. In one patient who was started under allopurinol therapy before transplantation, no evidence of crystal deposition was seen on biopsy performed four months after transplantation [31].

High doses of allopurinol may be necessary to treat or prevent DHA nephropathy in some patients. In the series by *Nasr et al*, DHA nephropathy relapsed in one patient receiving 300 mg per day of allopurinol. The allopurinol dose was increased to 600 mg per day and the patient had a good outcome with stable renal function and few crystals in renal biopsy [31]. We recommend starting allopurinol at 300 mg daily in most adult patients with normal body weight and monitoring crytalluria to guide treatment. Dosing must be adjusted when GFR is lower than 20 ml/min per 1.73 m^2^.

#### Surveillance

Repeated quantitative analysis of crystalluria is useful to guide treatment of APRT deficiency. A sustained fall of crystalluria is expected under treatment [6, 14]. The dose of allopurinol may be titrated upward if adequate response is not obtained with the initial dose. In this situation, it is also important to verify patient adherence to treatment.

### Prevention and management of recurrence of crystalline nephropathy in primary hyperoxaluria

Renal replacement therapy is eventually required in many patients with type 1 or 2 primary hyperoxaluria.

However, oxalate removal by dialysis cannot keep pace with the high generation rate of oxalate and prevent growing total oxalate burden [51]. Patients with advanced kidney disease, especially those on dialysis, are therefore at high risk to develop debilitating and life-threatening complications related to systemic oxalosis. For this reason, organ transplantation is far preferable to dialysis. Whenever possible, preemptive organ transplantation should be considered before CKD G4 (ie, when the GFR is still above 30 ml/min/1.73 m^2^). However, preemptive transplantation is not always possible and dialysis may be required. In such cases, intensified dialysis should be used to maximize oxalate removal. We, and others, recommend initiating dialysis early, when GFR is between 20 and 30 ml/min/1.73 m2, with the aim to maintain plasma oxalate below 50 μmol/l [52]. Oxalate is removed efficiently by hemodialysis, and to a lesser extent by peritoneal dialysis. However, considerable oxalate rebound occurs after each hemodialysis session [53, 54] so that 5 to 7 sessions per week using large dialyzers with high blood flows represent the most efficient way to remove oxalate in patients awaiting kidney transplantation [54]. The combination of hemodialysis and peritoneal dialysis may also be considered [52, 54].

As discussed above, kidney transplantation alone does not correct the underlying metabolic disorder. Other therapeutic measures are needed to prevent or treat the recurrence of crystalline nephropathy on the renal allograft.

#### Liver transplantation

In most cases, liver transplantation is the preferred therapeutic strategy for primary hyperoxaluria type 1 and represents the only way to stop the overproduction of oxalate. Of note, this does not apply to primary hyperoxaluria type 2. Although GRHPR enzyme is predominantly expressed in the liver [55] and isolated kidney transplantation is at risk of poor graft outcome [37], liver transplantation has not been reported in primary hyperoxaluria type 2. Combined liver-kidney transplantation should be considered on a case-by-case basis in primary hyperoxaluria type 2.

In primary hyperoxaluria type 1, preemptive liver transplantation alone may be considered in selected patients with relatively preserved kidney function (GFR >30 ml/min/1.73 m^2^) although the risk of death associated with this procedure must be weighed against the benefits [43]. In patients with more advanced kidney disease, liver-kidney transplantation represents the therapy of choice. In one study, 3-year death-censored kidney graft survival was 95 % in liver-kidney transplant recipients versus 56 % for those who received kidney transplantation alone [35]. In another study, kidney graft survival at 1, 3, and 5 years was 82 %, 79 %, and 76 %, respectively, for dual transplantation versus 46 %, 28 %, and 14 % for isolated kidney transplantation [36]. Interpretation of these data should be tempered by the likelihood that in these observational studies, the data are likely confounded by indication, with patients in better health receiving the scarce resource of liver transplantion. Since kidney transplantation alone is generally associated with poor outcomes, it may be considered only in selected adult patients with confirmed pyridoxine responsiveness (see *pyridoxine therapy* below) [56]. Although combined liver-kidney transplantation may be challenging in small children, good results may also be achieved in this population [57, 58]. Although liver transplantation corrects the enzymatic deficiency, severe hyperoxaluria can persist for several months or even years, because oxalate is mobilized from tissue stores [33]. The risk is particularly high if a long period has elapsed between the onset of renal failure and liver kidney transplantation. For this reason, sequential transplantation, with liver followed by kidney transplantation after a few months, may be an option in CKD G5 patients [59]. Two-step liver and kidney transplantation from a single living donor has been reported, with excellent outcomes for both donor and recipient [60]. Intensified peritoneal dialysis or hemodialysis strategies may be implemented to decrease the systemic oxalate burden during the interval between liver and kidney transplantation.

#### Pyridoxine therapy

Pyridoxine (vitamin B6) is a cofactor of the AGT enzyme. Administration of pyridoxine (starting dose 5 mg/kg daily) can decrease urine oxalate excretion in about one-third of patients with primary hyperoxaluria type I [4, 5]. It is recommended to test pyridoxine responsiveness in all patients with primary hyperoxaluria type 1 for at least 3 months and to continue therapy in all responsive patients [43]. Pyridoxine therapy is unnecessary after liver transplantation.

Some mutations (Gly170Arg and Phe152Ile) are associated with pyridoxine responsiveness [56]. In a small subset of patients, those homozygous for Gly170Arg, pyridoxine therapy may lead to normalization of urine oxalate levels. These patients may have excellent outcome after kidney transplantation alone [61]. However, in a recent study investigating responsiveness to pyridoxine therapy, patients homozygous for Gly170Arg did not achieve complete biochemical remission [62]. In addition, pyridoxine responsiveness can be difficult to assess in patients with advanced CKD. Thus, kidney-alone transplantation in patients homozygous for Gly170Arg remains controversial. Kidney-alone transplantation with long-term pyridoxine therapy may be considered on a case-by-case basis in Gly170Arg homozygous patients. This strategy may be a good option when liver transplantation is not possible, or when complete and sustained response to pyridoxine therapy could be clearly demonstrated prior to transplantation (normalization of urine oxalate levels after introduction of pyridoxine therapy).

#### Supportive measures

As discussed above, high urinary oxalate excretion can persist for a long time even after liver transplantation and jeopardize the success of kidney transplantation. Supportive measures aimed at decreasing urinary calcium-oxalate supersaturation are thus indicated in all kidney transplant recipients, including those receiving combined liver kidney transplantation. These measures should be immediately implemented in cases where primary hyperoxaluria is diagnosed post transplantation.

High fluid intake (>2 - 3 litres per m^2^ per day) is of paramount importance. A nasogastric tube or gastrostomy may be necessary to achieve this goal in children. Careful attention should be paid to any situation that could cause hypovolemia or decreased urine output. Citrate, which may be administered as potassium or sodium citrate, is also recommended to reduce urinary supersaturation of calcium-oxalate and crystallogenesis. Citrate acts by forming soluble complexes with calcium and by inhibiting crystal aggregation and growth [63]. Thiazide diuretics may also be helpful by increasing urine output and decreasing calciuria. In one study on children with hyperoxaluria after liver-kidney transplantation, the combination of high fluid intake, thiazides and citrate represented the most effective therapy to reduce crystal formation [41]. The benefit of post-transplantation dialysis to remove oxalate in an attempt to protect the renal allograft is controversial. Indications for post-transplantation dialysis are limited to patients with delayed graft function and in those with high systemic oxalate burden, who are particularly at risk for oxalate deposition in the new kidney. Although restriction of dietary oxalate is an effective treatment in primary hyperoxaluria, avoidance of oxalate-rich food seems sensible.

#### Surveillance

Serum oxalate levels rapidly fall to normal range in the days following liver-kidney transplantation and are not helpful in monitoring the risk of crystal formation [41]. Serial measurements of urinary oxalate and crystalluria are useful to guide the management of post-transplantation prophylaxis. Urinary oxalate excretion may be assessed in 24-hour urine collections or, more conveniently, in urine samples using the urine oxalate-creatinine ratio [5]. Quantitative analysis of crystalluria with oxalate crystal volume measurement in fresh urine samples may be helpful as it can be performed within hour and provides useful information for the guidance of fluid administration and prophylaxis against crystal formation almost in real time [41]. However, a standardized technique to quantify crystalluria is currently available only in some centres.

## Conclusions

Recurrence of crystalline nephropathy following kidney transplantation in patients with primary hyperoxaluria or APRT deficiency is an avoidable catastrophe. A high index of suspicion for these diseases should be maintained in all ESRD patients with a history of urolithiasis or crystals found in a native or transplant kidney biopsy. These patients should be investigated for APRT deficiency and primary hyperoxaluria. Favorable outcomes following allopurinol treatment or kidney transplantation, (in APRT deficiency) or liver-kidney transplantion (in primary hyperoxaluria) can be achieved.

## Abbreviations

APRT: Adenine phosphoribosyl transferase
DHA: 2,8 dihydroxyadenine
AGT: Alanine-glyoxylate aminotransferase
GRHPR: Glyoxylate reductase-hydroxypyruvate reductase (GRHPR)
HOGA: 4-hydroxy-2-oxo-glutarate aldolase
CKD: Chronic kidney disease
GFR: Glomerular filtration rate
ESRD: End-stage renal disease
